# Case of Obstruction of the Large Intestine, in Which the Ascending Colon Was Opened with Success, the Patient Dying Three Months Afterwards of Another Disease

**Published:** 1845-06

**Authors:** Samuel Evans

**Affiliations:** Derby


ROYAL MEDICAL AND CHIRURGICAL SOCIETY, APRIL 8.

*Case of obstruction of the Large Intestine, in which the Ascending Colon was opened with success, the Patient dying three months afterwards of another Disease.* By Samuel Evans, Esq. of Derby, communicated by Wm. Bowman, F. R. S., Asst. Surgeon to King's College Hospital.—Lewis S——, aged 23, a farmer, has been liable for several years to attacks of diarrhœa. In September, 1843, he was seized with violent pain in the bowels, resembling colic, which lasted for thirteen hours. About the third week in January, the attacks were renewed, and became more severe on the 5th of February. The author saw him for the first time on the 7th; he was suffering from severe intermitting pains in the abdomen, which was distended, but free from tenderness. There was a distinct swelling in the right iliac region. His bowels had not been relieved since the 5th. Opiates, active aperients, and stimulating injections were administered during five days without relieving the pain and sickness, or procuring evacuations. On the 12th and 13th, his sufferings were relieved by large doses of the liquor opii sedatives. From this time to the beginning of April, the size of his belly gradually increased; he also daily suffered many paroxysms of pain. At intervals large quantities of flatus and small portions of clay-coloured fæces escaped from the bowels. The patient's health became much impaired, and vomiting recurred almost daily. On the 25th of March, Callisen's operation, as modified by Amussat, for the formation of an artificial anus in the loin, was proposed, but the patient yielded to the wishes of his friends in postponing it. The emaciation increased, and the abdomen became distended to the greatest possible degree, the evacuations entirely ceased, and the pulse became feeble and fluttering. April 9th, the operation was performed; a transverse incision, four inches long, was made in the right loin; the ascending colon was opened, and more than two gallons of semi-fluid clay-coloured fæces were discharged. He recovered from the operation, and by May 9th had gained flesh, and the wound in the intestine had healed, but the evacuations escaped entirely by the artificial anus, being restrained by a plug in the orifice, which was removed four or five times a day. At the end of June, he commenced passing diabetic urine, and to suffer from thirst. July 2nd, he rode a distance of six miles in an uneasy cart, and shortly afterwards symptoms of peritonitis supervened, and he died on the fifth. On examination of the body, the cause of obstruction was found to be a stricture in the colon, just beyond the angle formed by the junction of the ascending and transverse portion of the gut. The contracted part was almost as hard as cartilage, and would only just admit a crowquill: its inner surface was ulcerated, the cæcum was enormously distended, and nearly as large as a stomach of ordinary size; the ascending colon was also much enlarged.

The author remarks that this is the eleventh case on record in which Callisen's operation (modified by Amussat) has been performed in the adult, in consequence of obstruction in the intestinal canal. From the previous history of the case, it would appear that the disease had been of slow progress, and of considerable duration ; but at the period to which the operation was delayed, owing to the interference of the patient's friends, he was in so alarming a condition that it is impossible to imagine a case more unfavourable for the operation. Two months afterwards, he was so much recovered that there appeared to be every prospect of his restoration to health, but these hopes were disappointed by his imprudence in regard to diet and exercise ; but as far as the operation is concerned, the case was successful.

Sir George Lefevre remarked on the indiscretion of the patient in going about so soon after the operation, and thought this should have been prevented by the medical attendant. Local and general treatment, together with the recumbent position, and strict attention to diet, were essential to the satisfactory conclusion of such a case. He briefly referred to two cases, of which he had drawn up a description, and had intended to read them to the Society, but had since withdrawn them, and published them elsewhere. In one of these cases, a lady of distinction in St. Petersburg had had the colon punctured merely for the purpose of relieving the pain caused by distention. In this case, the suffering was extreme ; there was incessant vomiting and most urgent distention. A trochar was plunged into the colon, an escape of gas took place, and relief ensued. She died, however, in eighteen hours afterwards. In another case of internal obstruction, the patient survived the operation twenty hours. Would an operation of this description be considered justifiable in this country, merely with a view of relieving symptoms, the termination of the case being certainly either way fatal ?

Mr. B. Philips regarded the paper as a very important one. There was no question that the operation had been performed many times with various modifications, both for obstructed bowels and imperforate anus. As far as he knew, it had been generally unsuccessful. The operation itself was by no means a difficult one, the real difficulty in these cases being to determine the circumstances which justify the proceeding. On what did the obstruction depend ? Cases of obstruction presented themselves, dependent on the collection of hardened fæces, in which the patient became emaciated, and the symptoms presented all the appearance of internal strangulation, yet these cases were relieved without operation. If the obstruction were the result of the disease in the rectum,—as, for instance, carcinoma, of which disease Broussais was said to have died,— here would be little difficulty in detecting its nature, and an operation like that under consideration might be performed with the chance of prolonging life. But even here we only substituted one infirmity for another, and it might be questionable which was the most difficult to be borne with. It was when the obstruction was situated higher up that it was difficult to decide on what it was dependent, and in what way we should proceed. The contraction, in fact, might exist at the very point at which the operation was usually performed. In Mr. Evans's case there was no indication presented to warrant us in drawing a conclusion as to the precise cause of the obstruction. Indeed, from the occasional passage of clay-coloured fæces, it might have been diagnosticated as arising from a fæcal accumulation, instead of being, as it was afterwards found, a stricture of the colon.

Dr. Powell remarked, that it was surprising how long constipation might exist in hysterical patients. He related the case of a lady who was constipated for three weeks, and who was relieved by the use of opium and croton oil. On one occasion she had no evacuation for two months. Injections were of no avail, and she took as much as half a grain of morphia and two drops of croton oil night and morning. In this case he believed the obstruction to be dependent merely on hysteria.

Mr. Davis related the case of a man who had been invalided from the West Indies, and whose bowels were moved only once in three weeks. His health was good, but he was occasionally subject to spasmodic pain in the abdomen. Extract of colocynth with opium was given without benefit, and he then took a compound gamboge pill three times a day, with small doses of Epsom salts. This usually succeeded every three weeks in procuring an evacuation, containing a great number of scybalæ. The only unpleasant symptoms under which he laboured were occasional attacks of spasmodic colic. Under this treatment his appetite improved, though he would now and then reject his food. He detailed another case of constipation following parturition, in which an evacuation, when it was obtained, was found to contain a great number of scybalæ. He mentioned these cases as illustrative of the effects of proper treatment in prolonged constipation.

Mr. Solly agreed with Mr. Philips as to the difficulty of determining the cause of obstruction in cases of prolonged constipation. Every surgeon in practice must have experienced this difficulty. He recollected a case which occurred some time since, of a woman who was tapped for what was supposed to be ascites, but which was found, after death, to be simple distention of the colon, with fæces, the result of schirrus of the rectum. The operation was followed by fatal peritonitis. He particularly referred to those cases of internal obstruction which resulted from the passage of adhesive bands from one portion of intestine to another. Some years since, he had seen a case of this description with Dr. Sutton, of Greenwich. He, Mr. Solly, had been called on to pass a rectum bougie in a case of obstinate constipation. This was readily done, no impediment to its passage being encountered. The patient died four days afterwards. After death a band of adventitious membrane was found extending from the colon to the mesentery, completely binding down the former, and obstructing its canal.

Dr. James Johnson considered that in Mr. Evans's case there was little difficulty in deciding at what point the obstruction existed. The fact of the bougie having readily passed, combined with the ready injection of three pints of fluid, and the position of the distention, indicated that the obstruction was near the caput coli. The operation he considered to have been perfectly justifiable. It was astonishing how long fæcal accumulations might exist, without seriously affecting the general health. He had a patient at present under his care, who had not passed any fæces by the rectum for the last three months. He was suffering from a large inelastic tumour near the caput coli, and daily vomited up fæcal matter. His appetite was good, his general health unaffected, and there did not appear to be any signs of a fatal termination. It might be a question how far an operation in this case might be advisable.

Mr. Dunn related the case of a child who was born with imperforate bowel, the obstruction of being situated so high up, that a bougie could not reach it. No operation was performed, and, after death, the colon was found to be not larger than a crowquill.

Mr. Blizard Curling observed that it was by no means an easy matter to reach the colon by operation, particularly in infants, and when that canal was undistended. He related a case in which this was attempted; the surgeon, however, cut down upon the kidney. In a case of imperforate anus, in which attempts to reach the rectum were fruitless, he advised the operation of Amussat, but it was not acceded to. After death, however, he, Mr. Curling, performed the operation, and found it less easy than some gentlemen would appear to think it. By cutting a little too near the spine, he came upon the kidney, an accident which he believed had occurred on more than one occasion when the operation had been performed on the living body. When the colon was distended, and the incision was made a little more externally, there would be no difficulty in reaching the intestine. He agreed with Dr. Johnson, that in Mr. Evan's case the cause of the obstruction was sufficiently evident to justify the operation. This case, indeed, offered a sufficient encouragement to other surgeons to resort to this proceeding, particularly in those cases in which the obstruction was as evident as in this instance.

Dr. Taylor remarked, that as Mr. Evans was clear in his opinion as to the seat of the obstruction in this case, the operation was justifiable, and had, undoubtedly, prolonged the life of his patient. There was nothing in the paper to show the nature of the obstruction, whether it was carcinomatous or otherwise. The microscope probably would be the only means of deciding this. It would appear, however, from the history of the case, that it originated in simple inflammation. It appeared that this patient had for some years been subject to diarrhœa; this was probably the result of inflammation, which had afterwards proceeded to ulceration, cicatrization, and contraction. He had seen similar cases of contraction from ulceration the result of fever, though not always situated in the same spot. In most of these cases no operation had been contemplated, as the patients had died from chronic peritonitis before the obstruction had continued sufficiently long to warrant any operative procedure. He thought that one mode of distinguishing whether the obstruction depended simply on the accumulation of hardened fæces, or on structural disease, would be to determine whether the patient had previously laboured under inflammation of the bowels. If so, the obstruction was probably structural. He had seen these obstructions in the small intestine frequently follow fever, they were the result of ulceration, cicatrization, and subsequent contraction.

Mr. Hilton considered that in Mr. Evans's case, the treatment, with respect to the operation, had been most judicious. The position of the obstruction was obvious, and the operation was therefore justifiable. He referred to cases of obstruction in the intestines resulting from a twisting of the colon upon itself, and of which he had lately seen an instance. The operation of Amussat was proposed, but not performed. After death, a vertical incision was made in the line of the abdominal muscles, from the false ribs to near the crista ilii; there was no difficulty in reaching the colon by this proceeding. The cases mentioned by Sir George Lefevre, in which the peritoneum was punctured, were very different from those under consideration, in which it was the object of the operator to avoid wounding that membrane.

Dr. Watson, from all he had seen or heard on the subject, had come to the conclusion, that the question of the operation was to be decided by the special circumstances of each case. In a case like that of Mr. Evans, the operation was justifiable, provided the patient had been acquainted with the nature and consequences of the proceeding previously. Other cases might occur, like that related by Dr. Johnson, in which, however inconvenient, it would, he considered, be advisable to allow fæcal evacuations by the throat, rather than resort to the formation of an artificial anus. Other cases would occur in which it could not be possible, á *priori*, to decide whether an operation were advisable or not.—*London Lancet.*

